# Size matters: nest colonization patterns for twig-nesting ants

**DOI:** 10.1002/ece3.1555

**Published:** 2015-07-17

**Authors:** Estelí Jiménez-Soto, Stacy M Philpott

**Affiliations:** Environmental Studies Department, University of California1156 High Street, Santa Cruz, CA, 95064

**Keywords:** Artificial nest, biodiversity ecosystem function, coffee agroecosystem, community assembly, niche partitioning

## Abstract

1. Understanding the drivers of ant diversity and co-occurrence in agroecosystems is fundamental because ants participate in interactions that influence agroecosystem processes. Multiple local and regional factors influence ant community assembly.
2. We examined local factors that influence the structure of a twig-nesting ant community in a coffee system in Mexico using an experimental approach. We investigated whether twig characteristics (nest entrance size and diversity of nest entrance sizes) and nest strata (canopy shade tree or coffee shrub) affected occupation, species richness, and community composition of twig-nesting ants and whether frequency of occupation of ant species varied with particular nest entrance sizes or strata.
3. We conducted our study in a shaded coffee farm in Chiapas, Mexico, between March and June 2012. We studied ant nest colonization by placing artificial nests (bamboo twigs) on coffee shrubs and shade trees either in diverse or uniform treatments. We also examined whether differences in vegetation (no. of trees, canopy cover and coffee density) influenced nest colonization.
4. We found 33 ant species occupying 73% of nests placed. Nest colonization did not differ with nest strata or size. Mean species richness of colonizing ants was significantly higher in the diverse nest size entrance treatment, but did not differ with nest strata. Community composition differed between strata and also between the diverse and uniform size treatments on coffee shrubs, but not on shade trees. Some individual ant species were more frequently found in certain nest strata and in nests with certain entrance sizes.
5. Our results indicate that twig-nesting ants are nest-site limited, quickly occupy artificial nests of many sizes, and that trees or shrubs with twigs of a diversity of entrance sizes likely support higher ant species richness. Further, individual ant species more frequently occupy nests with different sized entrances promoting ant richness on individual coffee plants and trees.

## Introduction

A central aim in ecology is to understand how diverse factors at local and regional scales influence community assembly. Community assembly is the process that leads to particular patterns of colonization of interacting (or not interacting) species, that may share a particular resource (HilleRisLambers et al. 2012), and a process that reflects survival of species in a particular habitat. The study of communities and their assemblage processes is important for explaining community dynamics, but also because it can provide important insights into spatiotemporal factors that maintain ecosystem services in face of global change, destruction of natural biomes, and intensification of managed systems (Philpott 2010; HilleRisLambers et al. 2012). Ants are a diverse and an interesting group of insects to use for studies of community assembly and drivers of coexistence because they are found almost everywhere and in the tropics they can represent up to 80% of the animal biomass (Hölldobler and Wilson 1990).

Understanding drivers of ant diversity and co-occurrence is of relevance, as ants participate in competitive, mutualistic and predatory interactions, as well as trait-mediated interactions that often result in ecosystem services (Liere and Larsen 2010; Vandermeer et al. 2010; Sanabria et al. 2014; Wielgoss et al. 2014). Ants are important pollinators (De Vega et al. 2014), predators of pests in agricultural systems (Vandermeer et al. 2010), seed dispersers (Lubertazzi et al. 2010), and protectors of plants that provide resources useful for ants (Rezende et al. 2014).

Local and regional factors influence ant assemblages; however, there is no single cause or dynamic that explains nest colonization patterns of entire communities of ants. Thus, recognizing that community assemblages can be structured through multiple ecological and evolutionary processes interacting synergistically is essential in community studies (Webb et al. 2002; Resetarits et al. 2005; Debout et al. 2009). By examining the community of arboreal ants that nest in hollow twigs in a coffee plantation, we investigated how availability of resources, such as diversity of nests with different sized entrances, and the vegetation strata in which nests are located influence colonization and nesting patterns for a community of twig-nesting ants. The role of cavity entrance diversity on Neotropical arboreal ants has been previously shown to strongly influence cavity colonization in a natural ecosystem (Powell et al. 2011). Although this study shares a number of similarities with the previous study in terms of the experimental design, the novelty of our study lies in the examination of the assembly process of the arboreal ant community in an agroecosystem considering the vegetative strata (and not canopy connectivity) as a potentially significant local factor influencing ant assembly.

Other studies have also made important contributions to the understanding of the influence of resource availability, interspecific competition from dominant ants, and changes in environmental conditions on ant colonization, survival, and community assembly (Ribas et al. 2003; Philpott 2010); similarly, studies have reported that niche differentiation and interspecific competition for similar resources structure ant communities (Albrecht and Gotelli 2001; Donoso 2014; Houadria et al. 2014). In the litter environment, factors such as patchiness in nest-site availability (but not necessarily availability of food resources) can influence ground ants (Kaspari 1996). For other communities, however, nesting sites might not be a limiting factor, although nest-site limitation may increase with agricultural habitat intensification or disturbance (Philpott and Foster 2005). Moreover, increases in diversity of nesting sites can influence species richness and composition (Armbrecht et al. 2004). Only few studies examine factors that influence ant communities at the colonization stage, despite the importance of priority effects for community assembly (Palmer et al. 2003; Andersen 2008; Livingston and Philpott 2010; Powell et al. 2011). Recruitment limitation can affect colony density and incidence of less competitive species, thus examining initial phases of colonization may be important for understanding species coexistence (Andersen 2008). Moreover, the dispersal stage of colony formation maybe strongly influenced by community assembly mechanisms such as habitat filtering because ants must find suitable habitats (Livingston and Philpott 2010).

This study asked the following questions: (1) Does nest strata or diversity of nest entrance sizes influence the percent of nests colonized by arboreal twig-nesting ants; (2) Does nest strata or diversity of nest entrance sizes influence the species richness of arboreal twig-nesting ants colonizing nests? (3) Does nest strata or diversity of nest entrance sizes influence the community composition of twig-nesting ants colonizing nests? (4) Are nests with certain nest entrance sizes more frequently occupied, or have a higher species richness of ants? (5) Do individual ant species more frequently occupy nests in a certain strata or nests of a certain entrance size?

## Methods

### Study site description

We conducted field research in Finca Irlanda (15°11′N, 92°20′W), a large, 300-ha shaded coffee farm in the Soconusco region of Chiapas, Mexico, between March and June 2012. The farm is located between 900 and 1100 m a.s.l. Between 2006 and 2011, annual rainfall at the farm was between 4000 and 5000 mm. During the time of the research, the production style of the farm could be classified as a mix of commercial polyculture and shaded monoculture according to the system of Moguel and Toledo (1999). The farm has ∼50 species of shade trees that provide 30–75% canopy cover to the coffee buses in the understory.

We studied ant occupation of nests in 44 locations (hereafter “sites”) on the farm. Each study site was separated by a minimum of 100 m and consisted of two neighboring *Inga micheliana* trees of approximately the same size (separated by 10–15 m) and two coffee plants directly underneath the trees. In order to characterize the vegetation of each study site, we measured trees, canopy cover, and coffee density. For all measurements, we used the midway point between the two *Inga micheliana* trees as the center point. In a 25 m radius circle around the center, we identified and counted each tree and measured the circumference and height of all tress. We sampled canopy cover at the circle center, and 10 m to the N, S, E, and W of the circle center with a convex spherical densitometer. We counted the number of coffee plants within 5 m of each focal *Inga* tree in each site. With the vegetation data, we calculated a vegetation complexity index (VCI). To calculate the index, we divided values for each vegetation variable (mean canopy cover, tree richness, mean tree height, mean tree circumference, percent of trees in the genus *Inga*, mean number of coffee plants) by the highest observed value for each variable. For the number of coffee plants and the percent of trees in the genus *Inga*, we subtracted the product from 1 as these two factors generally negatively correlate with vegetation complexity. Then, we took the average of all values for each site to obtain a single value between 0 (low vegetation complexity) and 1 (high vegetation complexity).

### Artificial nests and ant sampling

In each site, we added artificial nests to study nest colonization, following a similar methodology used by Powell et al. (2011). Artificial nests consisted of hollow bamboo twigs of the same cavity size (100 mm long, 10 mm internal diameter). We cut bamboo twigs such that the natural node blocked one end, and we plugged the other end of the bamboo with wood putty. We drilled circular holes (entrances) of the following sizes in the side of the bamboo: 1, 2, 4, 8, 16, and 32 mm^2^. The set of sizes used in the present study correspond to an exact subset of the cavity sizes used in Powell et al. (2011) – we did not use the largest size used in the previous study. We added six nests to each *Inga* tree and each coffee plant for a total of 24 nests added in each site, or 1056 nests added overall. In each site, we added a diverse mix of nests (one nest each of 1, 2, 4, 8, 16, and 32 mm^2^ nest entrance sizes) to one *Inga* tree and one coffee plant. On the other *Inga* tree and coffee plant, we added a uniform selection of nests (six nests all of the 32 mm^2^ nest entrance size). Treatments were randomly assigned to plants in each site. We attached nests to plants with twist ties and plastic string between 0.5 and 1.5 m above ground on coffee plants, and between 4 and 6 m above ground for *Inga* trees. We placed nests flush with coffee or tree branches. We placed nests between 5 and 7 March and harvested all nests 14 weeks later (between 14 and 18 June). The period of the study encompassed part of the rainy season. Rain and moisture have a significant effect on colony phenology because they regulate alate's flights in the absence of temperature variation (Kaspari et al. 2001). Although nests were placed long enough to be colonized by ants, longer time periods may have allowed us to capture colonization dynamics across time.

To determine effects of nest entrance size, entrance size diversity, and nest vegetation strata on colonization, we collected artificial nests, placed them in bags, froze them, and then cut open all nests to remove the contents. We noted whether each nest was occupied or not. We stored ants in 70% ethanol and later identified them according to the Ants of Costa Rica (Longino 2014) and AntWeb (2014). For all species found, we obtained an approximate head width measurement from AntWeb (2014).

### Data analysis

To compare whether the proportion of occupied nests differed with nest strata or the diversity of nest entrance sizes available, we used generalized linear mixed models (GLMM) with “glmer” in the “lme4” package in R (R Development Core Team 2014). We compared two models. In the first, we included nest strata (tree or coffee), nest size treatment (diverse or uniform), and the interaction between the two as fixed factors, the vegetation complexity index (VCI) as a covariate, and site as a random factor. In the second, we removed the VCI. To select the best model, we used the Akaike's Information Criterion (AIC) computed with the “mass” package (Venables and Ripley 2002). For both models, we used the binomial error distribution with the logit link. Instead of using the proportion data directly, we used the “cbind” function with number of nests occupied and number of nests that were not occupied as input variables.

To examine whether species richness differed with nest strata or the diversity of nest sizes available, we used two methods. First, we compared the mean species richness of ants occupying nests on a plant with GLMMs with “glmer” in the “lme4” package in R (R Development Core Team 2014). We compared two models. In the first, we included nest strata (tree or coffee), nest size treatment (diverse or uniform), and the interaction between the two as fixed factors, the vegetation complexity index (VCI) as a covariate, and site as a random factor. In the second, we removed the VCI. To select the best model, we used the Akaike's Information Criterion (AIC) computed with the “mass” package (Venables and Ripley 2002). For both models, we used a Poisson error distribution with the log link. Second, we created sample-based species accumulation curves, scaled to the number of individuals, to compare richness in coffee plants vs. trees and diverse vs. uniform nest size treatment plants with EstimateS (Colwell et al. 2004). We used the number of ant colonies encountered instead of the number of individuals, as ants are social organisms and better captured by number of colonies (Longino et al. 2002). We examined curves for both observed species richness and plotted 95% confidence intervals to statistically compare species richness between treatments.

To compare whether community composition of ants differed with strata and with nest size treatment, we used two methods. We used nonmetric multidimensional scaling (NMDS) and analysis of similarities (ANOSIM) in PAST (Hammer et al. 2001) to visually and statistically compare species composition of the ants occupying nests in coffee vs. shade trees and in uniform vs. diverse nest treatments. The ANOSIM compares (a) the mean distance within groups to (b) the mean distance between groups and can statistically determine separation in species composition between the plots in different treatment groups. For the NMDS and ANOSIM, we used the Bray–Curtis similarity index as the similarity measure.

Finally, we examined whether common ant species more frequently colonized nests of a certain entrance size or vegetation strata. To compare whether nests with certain entrance sizes were more frequently occupied by ants, we used an ANOVA followed by a Tukey's test to compare the mean proportion of nests of each entrance size that were occupied. We only used data from the diverse treatment plants (where nests of different size entrances were equally available) to calculate differences in nest colonization. To compare whether certain ant species more frequently occupied certain nest sizes or strata, we performed chi-squared analysis which is recommended for categorical data and tests the likelihood that an observed distribution is due to chance (Rao and Scott 1981).

## Results

Vegetation in the plots was somewhat variable. There were between eight and 31 trees, three and 12 tree species, and 12.5 and 36.5 coffee plants in each site. Mean tree height ranged from 4.4 m to 12.7 m, canopy cover ranged from 9.4% to 86.2%, and the VCI ranged from 0.28 to 0.74.

We recovered 1030 of the 1056 nests that were placed. Overall, we found 33 species of ants that colonized nests, and 73% of nests overall were occupied. The most common ants collected were *Camponotus atriceps* (18.72% of occupied nests), *Dolichoderus lutosus* (12.48%), *Pseudomyrmex gracilis* (6.77%), *Crematogaster sumichrasti* (6.37%), *Camponotus brettesi* (5.84%), *Crematogaster carinata* (5.44%), *Cephalotes basalis* (5.04%), *Camponotus novogranadensis* (4.9%), *Camponotus striatus* (3.98%), and *Neoponera crenata* (3.45%). Information on numbers of queens, males, workers, larvae, and pupae found for each species is presented in Table 1.

**Table 1:** Mean number of workers, queens, larvae, pupae, and males (alates) found in artificial nests, literature reports on their reproductive flight phenology of collected species

Ant Species	Workers	Queens	Larvae	Pupae	Males	Reproductive flight phenology
*Camponotus atriceps*	25.11	0.11	10.60	21.66	0.83	
*Camponotus brettesi*	49.95	2.36	25.66	37.32	11.18	
*Camponotus novogranadensis*	55.30	0.64	15.88	21.30	2.70	
*Camponotus striatus*	56.70	5.07	18.43	29.20	9.43	
*Cephalotes basalis*	67.08	1.03	29.75	23.08	0.09	
*Crematogaster carinata*	247.12	0.07	74.54	60.61	0.00	
*Crematogaster sumichrasti*	179.23	7.13	45.77	63.67	0.42	
*Dolichoderus lutosus*	124.59	3.80	42.54	50.11	4.74	More alates found in Feb–June^1^
*Pachycondyla crenata*	11.12	1.40	4.40	7.72	0.52	
*Pseudomyrmex gracilis*	26.98	3.45	25.64	15.80	1.06	Queens found in March, May^2^

1 Data from malaise traps in forest habitat on Barro Colorado Island (Kaspari et al. 2001).

2 Data from pan traps in coffee habitat in Chiapas, Mexico (Philpott, unpublished data).

**Figure 1:**
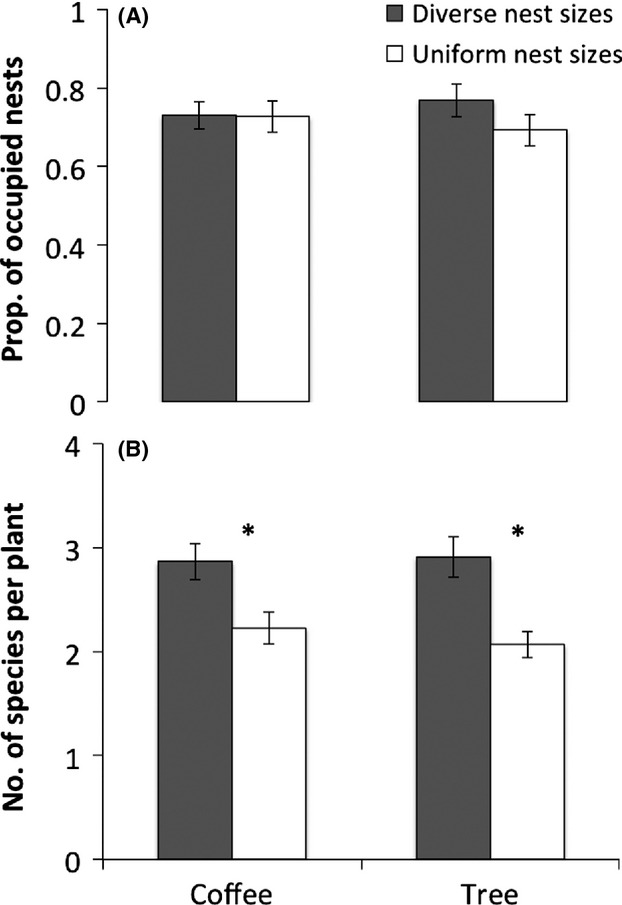
Influence of nest size entrance treatment (diverse and uniform) and vegetation strata (coffee and trees) on (A) the proportion of occupied nests and (B) species richness of ants colonizing nests. Asterisks show significant differences between nest entrance size treatments.

The proportion of occupied nests did not differ by nest strata or nest size treatment (Fig. 1A). The GLMM model that best predicted differences in the proportion of occupied nests included nest strata and nest size treatment as fixed factors and site as a random factor. Thus, although there was a large range in values for the vegetation characteristics measured and the VCI, vegetation complexity did not improve the model fit. There was no difference in the proportion of nests occupied in different nest size treatments (diverse and uniform) (*F*_1,43_ = 2.37, *P* = 0.131), or in different nest strata (*F*_1,43_ = 0.0112, *P* = 0.914), and there was no significant interaction between size treatment and strata (*F*_1,42_ = 1.948, *P* = 0.170).

**Figure 2:**
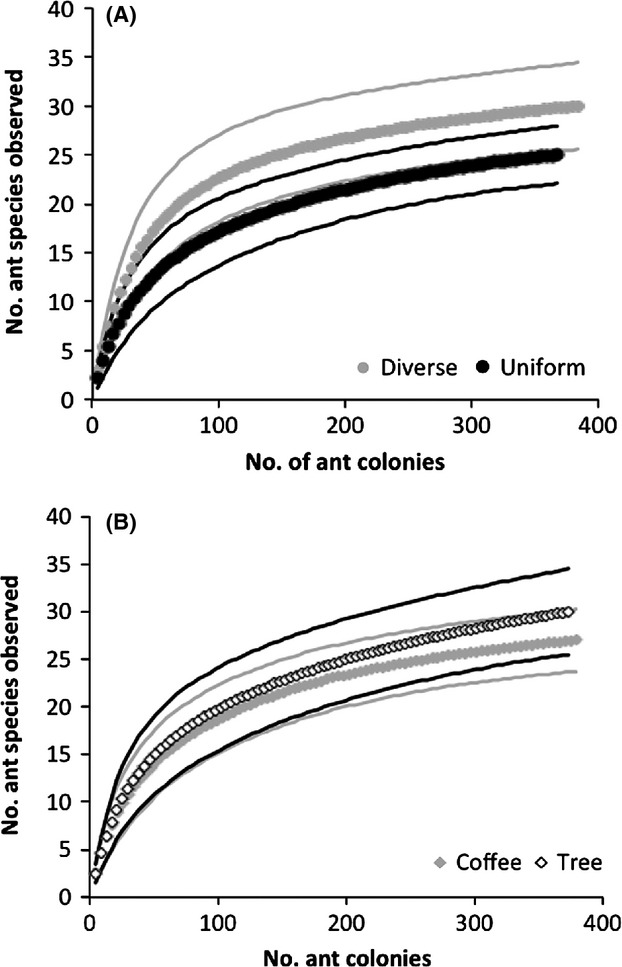
Species accumulation curves comparing ant species richness in (A) diverse nest size treatment nests (gray) and uniform nest size treatment nests (black) and (B) coffee nests (gray) and shade tree nests (open). Thick lines show richness and thin lines (of the same color) show 95% confidence intervals for observed and estimated richness.

Mean species richness increased with diversity of nest entrance sizes on a plant, but cumulative species richness did not differ between diverse and uniform treatment plants. The GLMM model that best predicted mean species richness included nest strata and nest size treatments as fixed factors and site as a random factor. Including the VCI did not improve model fit. The mean number of species on a plant was 20% higher on both coffee plants and shade trees with a diverse mix of nest sizes (*F*_1,43_ = 9.426, *P* = 0.004, Fig. 1B), but there were no differences in mean species richness with nest strata (*F*_1,43_ = 0.056, *P* = 0.814), and no significant interaction between size treatment and strata (*F*_1,42_ = 0.219, *P* = 0.643). In contrast, species accumulation curves did not show any difference in observed or estimated species richness between the diverse and uniform nest size treatments (Fig. 2A) or for coffee vs. shade tree strata (Fig. 2B).

**Figure 3:**
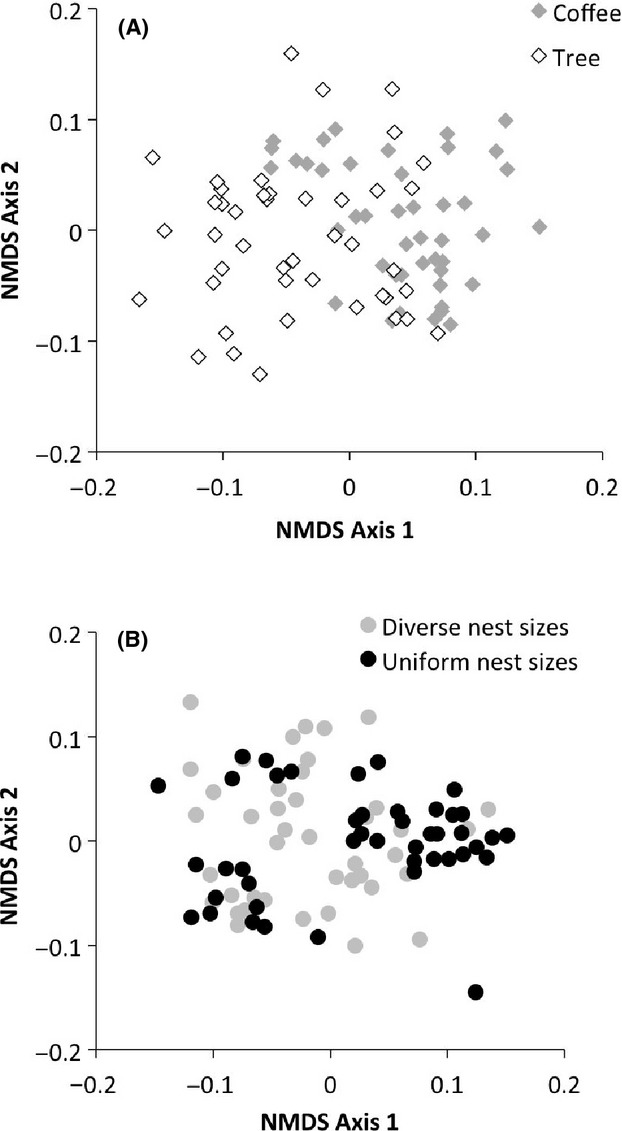
Nonmetric multidimensional scaling (NMDS) of the community of ants occupying (A) nests placed in coffee shrubs (gray) or shade trees (black) and (B) nests on plants with a diverse mix of nest entrance sizes (gray) or uniform nest entrance sizes (black).

Ant community composition of colonizing ants differed with both nest strata and nest size treatments. The NMDS for coffee and shade tree ant communities showed marked differences between the two nest strata (Fig. 3A, stress = 0.348), and the ANOSIM demonstrated a significant difference between the two groups of ants (Global *R* = 0.2475, *P* < 0.001). Likewise, the NMDS showed different ant community composition in the diverse and uniform nest size treatment plants (Fig. 3B, stress = 0.3316) and the ANOSIM showed a significant difference between ants in nests on diverse and uniform treatment plants (Global *R* = 0.1318, *P* < 0.001).

**Figure 4:**
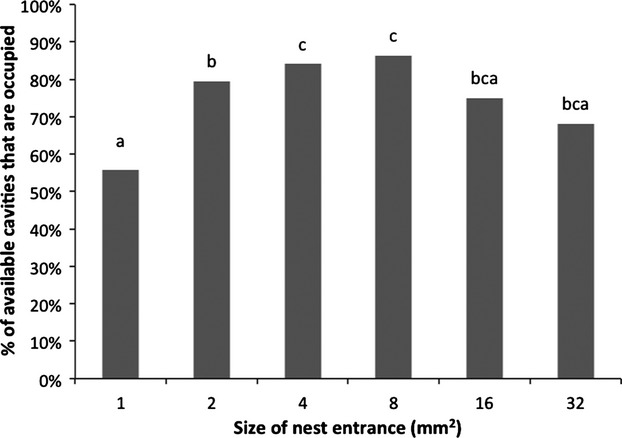
The percent of nests of each nest size entrance occupied by ants on the diverse size treatment plants. The numbers above each column show richness of ants in that nest entrance size, and small letters indicate differences in percent occupation in different nest sizes according to pairwise Tukey's tests (P < 0.05).

Ants more frequently occupied nests with certain entrance sizes and richness in different sizes also differed. Of all available nest sizes, the middle sizes were more frequently occupied (*F*_5,259_ = 19.05, *P* < 0.001, Fig. 4). There were pairwise differences in proportion of occupied nests for many pairs of entrance sizes (*P* < 0.05).

**Figure 5:**
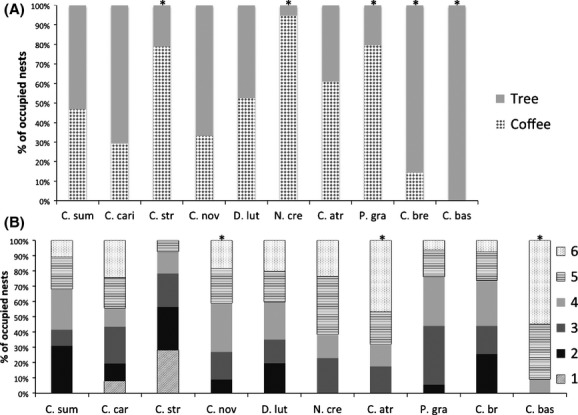
The frequency with which certain ant species [*Crematogaster carinata* (C. cari), *Camponotus striatus* (C. str), *Camponotus novogranadensis* (C. nov), *Dolichoderus lutosus* (D. lut), *Neoponera crenata* (N. cre), *Camponotus atriceps* (C. atr), *Pseudomyrmex gracilis* (P. gra), *Camponotus bretesi* (C. bre), and *Cephalotes basalis* (C. bas)] occupy (A) nests in the coffee and shade trees and (B) nests of different sized entrances. Significant differences in occupation of different strata or sizes are indicated with an asterisk.

The chi-squared analysis showed that certain ant species more frequently occupied nests with certain entrance sizes or placed in different vegetation strata (Fig. 5A and B). In particular, *P. gracilis* more frequently occupied nests with 4 mm^2^ entrances than nests with other entrance sizes (*χ*^2^ = 15.09, df = 5, *N* = 26, *P* = 0.0001; *C. basalis* more frequently occupied nests with the largest entrance size (32 mm^2^, *χ*^2^ = 12.37, df = 5, *N* = 10, *P* = 0.003), as did *C. atriceps* (*χ*^2^ = 11.07, df = 5, *N* = 20, *P* = 0.008). The other ant species did not more frequently occupy certain nest sizes. Likewise, half of the most common ant species found more frequently occupied nests in one of the two nest strata (Fig. 5B). Specifically, three species, *C. striatus*, *P. gracilis*, and *N. crenata,* more frequently occupied nests placed on coffee shrubs (*C. striatus*, *χ*^2^ = 6.63, df = 1, *N* = 19, *P* = 0.01; *P. gracilis*, *χ*^2^ = 13.56, df = 1, *N* = 39, *P* = 0.0002; *N. crenata*, *χ*^2^ = 15.21, df = 1, *N* = 19, *P* < 0.001). *C. bretesi* more frequently occupied nests in trees (*χ*^2^ = 10.82, df = 1, *N* = 28, *P* = 0.0001). *C. basalis* only occupied nests in trees (*χ*^2^ = 22, df = 1, *N* = 22, *P* < 0.001).

## Discussion

Ecological studies strive to understand local and regional factors that influence community assembly and species coexistence (Ricklefs 1987; Drake 1991; Huston 1999; Chesson 2000; Hubbell 2001; Chase 2003, Foster et al. 2004; Leibold et al. 2004; Powell et al. 2011). Some factors important for assembly of arboreal twig-nesting ants include the presence of a canopy dominant species (Philpott 2010) and resource access through canopy connectivity (Powell et al. 2011). Previous studies have found that diversity of nesting resources influences the colonization process of leaf-litter twig-nesting ants (Armbrecht et al. 2004) and of tropical arboreal ants (Powell et al. 2011) and that the abundance of nesting resources may impact colonization of arboreal twig-nesting ants (Philpott and Foster 2005).

The study of assembly in ant communities in a spatial context reveals that species sorting, by which different species specialize in a particular habitat, and mass effects, in which species disperse from less to more suitable habitats, are likely important for common and rare species, respectively, in agroecosystems – habitats embedded in landscape mosaics were local communities interact through dispersal (Leibold et al. 2004; Livingston et al. 2013). Our study is novel in that we examined colonization in a managed ecosystem looking at two factors (nest entrance size and strata) and their importance in colonization. In this study, we suggest that nesting resource utilization, specifically different frequencies of occupation of specific nest entrance sizes and specific nesting strata are important drivers of community assembly.

Overall, we found that nesting strata (shade tree or coffee shrub) and the diversity of nest entrance sizes (uniform vs. diverse treatments) did not significantly influence the proportion of occupied artificial nests. Thus, ants use newly available cavities for colonization and nesting resources are somewhat limiting for the community of twig-nesting ants in the habitat studied. In comparison with our study, Powell et al. (2011) found that total nest occupancy was higher with higher nest cavity diversity in the Brazilian savanna (3% occupation in uniform entrance size vs. 26% in diverse entrance). It is possible that such distinct results derive from differences in overall nest availability, differences in vegetation (e.g., coffee systems with abundant woody shrubs and trees with 30–75% canopy cover, Cerrado systems with a grass and shrub dominated ground and 30–50% canopy cover with trees up to 8 m) and differences in the abundance of particular genera (e.g., *Cephalotes*). However, it is important to consider that the near-saturation found in the present study could be a result of adding only one cavity of each size per plant, which could mean that there were not enough nests to be colonized, once the “preferred” sizes (mainly mid-size cavities) were used on every plant – hence not available for other species to occupy. Differences in nest saturation and the proportion of nest occupation between both studies could be due to differences in the number of cavities per size used in the experiment. Thus, it is difficult to say that differences in nest limitation are due to the agroecological context, as previous studies in coffee plantations have found that the community of twig-nesting ants are limited by nesting resources (Armbrecht et al. 2006), as are ants in natural ecosystems (Kaspari 1996; Powell et al. 2011). In addition, differences in occupation dynamics of artificial nests during the colonization phase could potentially change with length of the study. A clear contrast is that the present study lasted 3 months, a third of the previous study, this difference in time could potentially influence competition for “preferred” cavities during colonization, as these are available for a longer period of time during the colony life cycle. Very little information is available about the reproductive phenology of arboreal twig-nesting ants. The evidence collected from our nests indicates (Table 1) that all common species were producing larvae and pupae, and that most species nests did contain alate males. Two of the common species collected from nests in the present study do experience queen flights during this time period (Table 1), but information is lacking for the other species. Thus, timing of nest placement may have affected the colonization processes, but it is important to note that many twig-nesting species expand by colony budding, and not only nuptial flights. Changes in the occupation dynamics – that is, proportion of occupied nests, changes in diversity and species interactions – through time could be the focus of future studies.

Even though diversity of nest entrance sizes did not influence the percentage of occupation overall, frequency of occupation of nests by ants did differ for particular sizes. Higher occupancy was found in middle sizes (2, 4, 8 mm^2^), and these results are similar to Powell et al. (2011) in which middle sizes (4, 8, and 16 mm^2^) were the most frequently occupied. The specificity in the use of particular sizes is important in two ways: First, the evolution of ecological specialization underlies the evolution of morphological specialization in ant soldiers, Powell (2008) showed that for different species of *Cephalotes* an increase in ecological specialization (meaning the use of cavities that matched the size of one ant head) corresponded to a higher head specialization (head morphology); in that same study, *C. persimilis* uses cavities that match the size of one soldier's head and it has also evolved a highly specialized complete head-disk, while less ecologically specialized *Cephalotes* species, such as *C. pusilus* (occupying cavities as big as 10 ant head sizes) have evolved a domed-head. Second, such size specialization maximizes individual nest survival and is likely to have a positive effect on overall colony reproduction as shown previously for *C. persimilis,* which more frequently nests in cavities that fit its head size (Powell 2009). On the other hand, *Cephalotes* ants using cavities larger than their soldier's head allow them to protect the nest using cooperative blocking (Powell 2009). The present study supports the former hypothesis (that ecological specialization drives a specialized morphology) (Powell 2008), in that the *Cephalotes* species present in our study (*C. basalis*), a domed-headed soldier morphotype, was more frequently found in the largest size (32 mm^2^ area), an entrance size much larger than the ant's head maximum-recorded width (∼3.16 mm) (Table 2, de Andrade and Baroni Urbani 1999). Other *Cepahlotes* species (e.g., *pusilis*) prefer natural nest sizes between four and up to ten times their head size (Powell 2008). If *C. basalis* shows a similar preference, and if we assume a maximum head size of ∼5 mm (Powell 2008), then its preferred size might be between the 16 and 32 mm^2^ nests offered in this study.

**Table 2:** Head sizes of common ant species encountered and nest entrance size that was more frequently occupied by that ant. Frequencies indicated with an asterisk were statistically significant. Species are arranged from smallest to largest

Species	Approximate head size^1^	Nest entrance size more frequently occupied
*Crematogaster sumichrasti*	0.60 mm	2 mm^2^
*Crematogaster carinata*	0.67 mm	4 mm^2^
*Camponotus striatus*	0.75 mm	2 mm^2^
*Camponotus novogranadensis*	0.92 mm	8 mm^2^
*Dolichoderus lutosus*	1.25 mm	8 mm^2^
*Neoponera crenata*	1.42 mm	16 mm^2^
*Camponotus atriceps*	1.53 mm	32 mm^2^*
*Pseudomyrmex gracilis*	1.61 mm	4 mm^2^*
*Camponotus brettesi*	1.87 mm	8 mm^2^
*Cephalotes basalis*^2^	3.16 mm	32 mm^2^*

1 Head size represents the widest section of the head as obtained from AntWeb (2014).

2 Head size from de Andrade and Baroni Urbani (1999).

Mean species richness was not different in artificial nests on coffee plants and trees; however, the diversity of nest entrance sizes increased mean species richness on individual trees and coffee plants. In contrast to a previous study, in which diversity of nest cavities did not significantly affect the number of ant species per tree (Powell et al. 2011), we did find that providing a diverse array of twig entrance sizes promoted local (e.g., plant level) ant species richness in both coffee shrubs and shade trees. This supports the idea that making a diversity of resources available in both strata supports a more diverse mix of arboreal twig-nesting ants. That we found more species richness per tree (and not per site, shown by the species accumulation curves) when providing a higher diversity of nest sizes could indicate that competition for resources might happen more intensively at the local scale, rather than at larger spatial scales. Diversity of nest resources is important for other twig-nesting ant communities. Namely, in a study of leaf-litter twig-nesting ants in shade coffee plantations in Colombia, 80% more species were found when providing a diverse mix of twigs rather than a monospecific collection of twigs (Armbrecht et al. 2004) showing that diversity of twig-nesting ants is influenced by other aspects of diversity of nesting resources.

We found that certain ant species more frequently occupied particular sizes and this may be in part, an explanation for why we found higher species richness on individual plants with a diversity of nest entrance sizes. Armbrecht et al. (2004) showed the importance of a diverse mix of twigs for species richness; however, the driver in their study was not preference of different ant species for a different species of twigs, but rather an emergent property of a diverse mix of twigs. In our study, we provide evidence that species sorting along a size gradient likely explains the differences observed in mean species richness in uniform vs. diverse treatments. The frequency of occupation differed between sizes for certain ant species, largely following differences in ant head sizes (Table 2). As small ants can occupy a nest with a wide array of entrance sizes, larger ants can only occupy nests with entrances sizes larger than the workers. Thus, providing a wider diversity of nest sizes may allow for greater niche differentiation in the ant community. This outcome might increase the overall richness of the ant community or on individual plants. In our study, larger ants seem to be more size limited than smaller ants, likely because larger ants simply cannot fit into the nests with smaller entrance sizes, and thus are directly constrained by the availability of twigs that fit their body dimensions (Kearney and Porter 2009). In vastly different systems, similar properties operate. For example, in aquatic systems, water temperatures can limit temporal and spatial distribution of certain species as morphological constraints can significantly limit species' access to suitable habitats (Kearney and Porter 2009). Alternatively, models of exploitative competition (Tilman 1990) have suggested that when two species compete for one limiting resource the result of such competition is determined by the species more capable to attain the lowest equilibrium resource concentration possible, R* (Begon et al. 2006). In other words, R* becomes a factor that is the lowest extent to which a certain species can survive in a certain area.

Community composition varied between plants with uniform vs. diverse nest entrance sizes, as well as in coffee plants and shade trees. Our results are consistent with previous studies that have investigated ant stratification in the rainforest, where there is a strong partitioning of ant species in the leaf litter, lower vegetation, and canopy (Brühl et al. 1998). Likewise, tropical ant activity is often higher in the canopy than in the litter environment, and species composition differs between the canopy and litter assemblages (Yanoviak and Kaspari 2000). A study in natural ecosystems comparing forest and savanna found species richness to be affected by habitat and strata (ground and vegetation); the two environments clearly differentiated in terms of their species composition (Vasconcelos and Vilhena 2006). In our study, canopy vegetation was not a strong driver for the community of twig-nesting ants as our best models did not include a VCI. However, species compositional differences observed across both vegetation layers could be an effect of microhabitat diversity (Brühl et al. 1998) and canopy connectivity (Powell et al. 2011). Providing complex vegetation not only promotes ant diversity but also other organisms that facilitate ant colonization into new twigs. Presumably ants often nest in hollow branches of trees that have been previously dwelled or inhabited by beetles (Deyrup et al. 2000). Moreover, diversity of trees might also provide nesting resources that are different in terms of how difficult or attractive they are to dig cavities, for example, studies have found that tropical woods can be different in terms of their structure, chemistry and biology (Perez-Morales et al. 1977); this could suggest important drivers in the differentiation of ants that inhabit them.

We found a large number of arboreal twig-nesting ant species (33) in this coffee agroecosystem study supporting the notion that managed ecosystems, such as agroforestry systems in the tropics, have the potential to host a great diversity of species. A number of previous studies have provided evidence that ant diversity increases control of pests and fungal diseases (Philpott and Armbrecht 2006). We document here that increases in nest entrance size diversity on an individual tree relates to increases in ant diversity on trees. This may thus have important implications for promoting ants as biological control agents in agroforestry systems.

We conclude that the availability of a variety of nesting options (in this case different nest entrance sizes) and vegetation strata are important drivers of species diversity and support the idea that niche partitioning drives species coexistence (Chase and Leibold 2003). Future studies should further investigate the competitive hierarchies of the species colonizing twigs if we want to understand how species using similar resources interact with each other; and evaluate colony fitness in face of multiple resource use, as has been done in the past for colonies of *Cephalotes persimilis* (Powell 2009). As ants often engage in interactions that deliver ecosystem services future studies should focus on evaluating roles of different ant combinations using a diverse array of twig entrance sizes in agricultural pest control. Furthermore, we have learned from this study that the structuring of ant communities is multifactorial and that local as well as regional factors should be considered when explaining species assemblages in the tropics.
